# Agent-Based Modeling of Oxygen-Responsive Transcription Factors in *Escherichia coli*

**DOI:** 10.1371/journal.pcbi.1003595

**Published:** 2014-04-24

**Authors:** Hao Bai, Matthew D. Rolfe, Wenjing Jia, Simon Coakley, Robert K. Poole, Jeffrey Green, Mike Holcombe

**Affiliations:** 1 Department of Computer Science, University of Sheffield, Sheffield, United Kingdom; 2 Department of Molecular Biology and Biotechnology, University of Sheffield, Sheffield, United Kingdom; Johns Hopkins University, United States of America

## Abstract

In the presence of oxygen (O_2_) the model bacterium *Escherichia coli* is able to conserve energy by aerobic respiration. Two major terminal oxidases are involved in this process - Cyo has a relatively low affinity for O_2_ but is able to pump protons and hence is energetically efficient; Cyd has a high affinity for O_2_ but does not pump protons. When *E. coli* encounters environments with different O_2_ availabilities, the expression of the genes encoding the alternative terminal oxidases, the *cydAB* and *cyoABCDE* operons, are regulated by two O_2_-responsive transcription factors, ArcA (an indirect O_2_ sensor) and FNR (a direct O_2_ sensor). It has been suggested that O_2_-consumption by the terminal oxidases located at the cytoplasmic membrane significantly affects the activities of ArcA and FNR in the bacterial nucleoid. In this study, an agent-based modeling approach has been taken to spatially simulate the uptake and consumption of O_2_ by *E. coli* and the consequent modulation of ArcA and FNR activities based on experimental data obtained from highly controlled chemostat cultures. The molecules of O_2_, transcription factors and terminal oxidases are treated as individual agents and their behaviors and interactions are imitated in a simulated 3-D *E. coli* cell. The model implies that there are two barriers that dampen the response of FNR to O_2_, i.e. consumption of O_2_ at the membrane by the terminal oxidases and reaction of O_2_ with cytoplasmic FNR. Analysis of FNR variants suggested that the monomer-dimer transition is the key step in FNR-mediated repression of gene expression.

## Author Summary

The model bacterium *Escherichia coli* has a modular electron transport chain that allows it to successfully compete in environments with differing oxygen (O_2_) availabilities. It has two well-characterized terminal oxidases, Cyd and Cyo. Cyd has a very high affinity for O_2_, whereas Cyo has a lower affinity, but is energetically more efficient. Expression of the genes encoding Cyd and Cyo is controlled by two O_2_-responsive regulators, ArcBA and FNR. However, it is not clear how O_2_ molecules enter the *E. coli* cell and how the locations of the terminal oxidases and the regulators influence the system. An agent-based model is presented that simulates the interactions of O_2_ with the regulators and the oxidases in an *E. coli* cell. The model suggests that O_2_ consumption by the oxidases at the cytoplasmic membrane and by FNR in the cytoplasm protects FNR bound to DNA in the nucleoid from inactivation and that dimerization of FNR in response to O_2_ depletion is the key step in FNR-mediated repression. Thus, the focus of the agent-based model on spatial events provides information and new insight, allowing the effects of dysregulation of system components to be explored by facile addition or removal of agents.

## Introduction

The bacterium *Escherichia coli* is a widely used model organism to study bacterial adaptation to environmental change. As an enteric bacterium, *E. coli* has to cope with an O_2_-starved niche in the host and an O_2_-rich environment when excreted. In order to exploit the energetic benefits that are conferred by aerobic respiration, *E. coli* has two major terminal oxidases: cytochrome *bd*-I (Cyd) and cytochrome *bo′* (Cyo) that are encoded by the *cydAB* and *cyoABCDE* operons, respectively [1], [2]. Cyd has a high affinity for O_2_ and is induced at low O_2_ concentrations (micro-aerobic conditions), whereas Cyo has a relatively low affinity for O_2_ and is predominant at high O_2_ concentrations (aerobic conditions) [3]. These two terminal oxidases contribute differentially to energy conservation because Cyo is a proton pump, whereas Cyd is not [1], [2]; however, the very high affinity of Cyd for O_2_ allows the bacterium to maintain aerobic respiration at nanomolar concentrations of O_2_, thereby maintaining aerobic respiratory activity rather than other, less favorable, metabolic modes [4]–[6].

**Figure 1:**
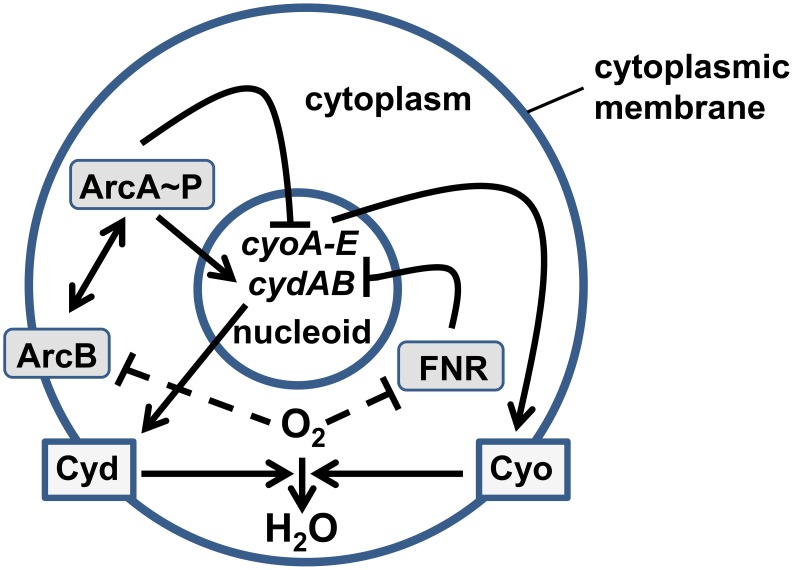
Components of the agent-based model. The diagram shows the interactions between and locations of the components of the model. Oxygen molecules (O_2_) cross the cytoplasmic membrane and enter the bacterial cell where they are reduced to water (H_2_O) by the action of the membrane-bound terminal oxidases (Cyo and Cyd). The transcription regulator, FNR is located in the cytoplasm and is inactivated by reaction with O_2_. The active form of FNR represses the expression of both the *cydAB* and *cyoABCDE* operons located in the nucleoid. The ArcBA two-component system responds to O_2_ indirectly (dashed line). The availability of O_2_ alters the redox state of the electron transport chain and the production of fermentation products. These changes are sensed by the membrane-bound sensor ArcB, which autophosphorylates when O_2_ is restricted. ArcB transfers phosphate to the cytoplasmic regulator ArcA, which acts to repress expression of *cyoABCDE* and activate expression of *cydAB*.

The transcription factors, ArcA and FNR, regulate *cydAB* and *cyoABCDE* expression in response to O_2_ supply [7]. FNR is an iron-sulfur protein that senses O_2_ in the cytoplasm [8], [9]. In the absence of O_2_ the FNR iron-sulfur cluster is stable and the protein forms dimers that are competent for site-specific DNA-binding and regulation of gene expression [10]. The FNR iron-sulfur cluster reacts with O_2_ in such a way that the DNA-binding dimeric form of FNR is converted into a non-DNA-binding monomeric species [10]. Under anaerobic conditions, FNR acts as a global regulator in *E. coli* [11]–[13], including the *cydAB* and *cyoABCDE* operons, which are repressed by FNR when the O_2_ supply is restricted [7]. Under aerobic conditions, repression of *cydAB* and *cyoABCDE* is relieved and Cyd and Cyo proteins are synthesized [3]. In contrast, ArcA responds to O_2_ availability indirectly via the membrane-bound sensor ArcB. In the absence of O_2_ ArcB responds to changes in the redox state of the electron transport chain and the presence of fermentation products by autophosphorylating [14]–[16]. Phosphorylated ArcB is then able to transfer phosphate to the cytoplasmic ArcA regulator (ArcA∼P), which then undergoes oligomerization to form a tetra-phosphorylated octomer that is capable of binding at multiple sites in the *E. coli* genome [17], [18], including those in the promoter regions of *cydAB* and *cyoABCDE* to enhance synthesis of Cyd and inhibit production of Cyo [7], [17]. Because the terminal oxidases (Cyd and Cyo) consume O_2_ at the cell membrane, a feedback loop is formed that links the activities of the oxidases to the regulatory activities of ArcA and FNR (Figure 1). These features of the system - combining direct and indirect O_2_ sensing with ArcA∼P and FNR repression of *cyoABCDE*, and ArcA∼P activation and FNR repression of *cydAB* - result in maximal Cyd production when the O_2_ supply is limited (micro-aerobic conditions) and maximal Cyo content when O_2_ is abundant (aerobic conditions) [3].

Although the cellular locations of the relevant genes (*cydAB* and *cyoABCDE*), the regulators (ArcBA and FNR) and the oxidases (Cyd and Cyo) are likely to be fundamentally important in the regulation of this system, the potential significance of this spatial organization has not been investigated. Therefore, a detailed agent-based model was developed to simulate the interaction between O_2_ molecules and the electron transport chain components, Cyd and Cyo, and the regulators, FNR and ArcBA, to shed new light on individual events within local spatial regions that could prove to be important in regulating this core component of the *E. coli* respiratory process.

## Results/Discussion

The starting point for this work was the suggestion that spatial effects play an important role in controlling the response of the *E. coli* transcription factor FNR to changes in O_2_ availability [15]. Early work showed that when dissolved O_2_ was detectable in the culture medium the activity of FNR decreased, exhibiting ∼50% activity in the range 2–5 µM dissolved O_2_ and that under these conditions the external concentration of O_2_ was equivalent to that in the bacterial cytoplasm [19], [20]. However, when the cultures were supplied with low concentrations of O_2_ in the input gas, dissolved O_2_ in the culture medium could not be measured conventionally, because the O_2_ is consumed by the respiratory activity of the bacteria. Therefore, a physiological measure of O_2_ availability (the aerobiosis scale) was adopted to investigate the effects of low O_2_ concentrations on bacteria [21]. On the aerobiosis scale, the minimum O_2_ input that results in undetectable excretion of the fermentation product acetate under carbon-limiting conditions is defined as 100% aerobiosis (100% AU). As the amount of O_2_ supplied to cultures decreases the specific rate of acetate production (*q*_acetate_) increases to a maximum under anaerobic conditions (0% AU). Between these limits (0–100% AU) lies the micro-aerobic range, defined by the linear decrease in *q*_acetate_ as the O_2_ transfer rate increases, i.e. there is an inverse correlation between *q*_acetate_ and aerobiosis [21]. When the O_2_ supply exceeds the minimum required to abolish acetate excretion the AU value extends beyond 100%, reaching 217% when the culture medium is O_2_ saturated. This physiological measurement of O_2_ availability is reliable for cultures grown at values as low as 4% AU [21]. In previous experiments using this approach steady-state chemostat cultures were established at fixed points on the aerobiosis scale and samples were taken for measurement of the numbers of Cyd and Cyo molecules per bacterial cell (Table 1) [3]. In addition, Western blotting showed that the concentration of FNR in the cell was constant at 3000 protomers per bacterium across the aerobiosis scale (Table 1).

**Table 1:** Numbers of Cyd, Cyo, ArcA and FNR molecules per *E. coli* cell at different points on the aerobiosis scale.

AU (%)	Cyd (molecules per cell)	Cyo (molecules per cell)	Total ArcA monomer (molecules per cell)	Total FNR monomer (molecules per cell)
0	11442	4336	8000	3000
31	51403	6202	8000	3000
85	66729	14017	8000	3000
115	19102	10985	8000	3000
217	10284	9036	8000	3000

The Cyd, Cyo and ArcA numbers are those reported by Rolfe et al. [3]. The number of FNR molecules per cell was calculated by from analysis of Western blots developed with anti-FNR serum for whole cell samples taken from steady-state cultures at the indicated aerobiosis units (AU).

**Figure 2:**
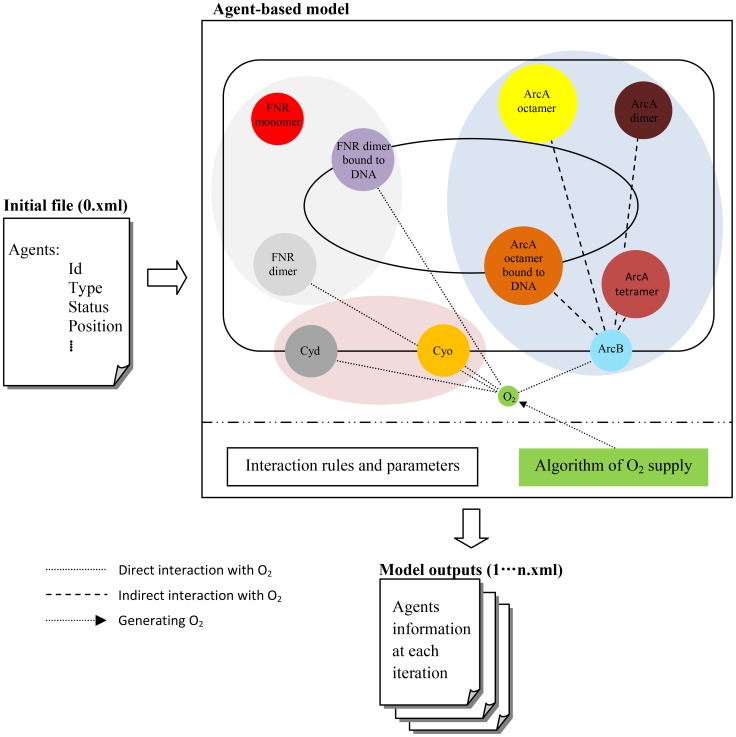
Components and process of agent-based model simulation. The file 0.xml contains all the settings for the agents involved to provide the initial model state. The FNR system, ArcBA system and terminal oxidases are displayed in light grey, light blue and light red ellipses respectively. The interaction rules and parameters are pre-defined, which determines how and when the interactions take place. For each of the iterations the number of O_2_ molecules supplied to modelled cell is calculated. While model runs, updated information is generated in the same format as the initial file and stored in a series of xml files for further analysis. Experimental data, where available, were used for preparing the initial file and designing the interaction rules and parameters.

The agent-based model was constructed to simulate interactions between three groups of agents: O_2_ molecules, terminal oxidases, (Cyd and Cyo) and regulators (FNR and ArcBA), which interact with O_2_ directly or indirectly. As shown in Figure 2, the initial state of the model is defined in the file 0.xml, which contains information on agent properties such as Id, type, status, position etc. – the data in Table 1 was used to provide numbers of Cyd, Cyo, ArcA and FNR molecules. There is no information available on the abundance of ArcB in the cell and thus it was assumed that there are 1000 ArcB molecules per cell based on the ∼10∶1 ratio of response-regulator to sensor kinase of another *E. coli* two-component system, PhoBR [22]. At the beginning of the simulation, the 0.xml file is read to establish the 3-D *E. coli* cell. From the initial state, the agents start moving randomly within their 3-D activity space, and interact with each other according to a set of pre-defined interaction rules and interaction radii (Tables 2 and 3), leading to an emergent state.

**Table 2:** Agent properties.

Molecule	Form	Initial number	Step length (nm)	Initial location	Activity space
O_2_	Molecule	0	Variable^1^	Outside the cell	From outside to inside of the cell
FNR	Monomer	0	15	n/a	Inside the cell
	Free dimer	1150	15	Evenly distributed in the cell	Inside the cell
	Dimer bound to DNA	350	0	Inside the nucleoid space	Stationary on DNA
ArcA^2^	Octamer	1000	18	Evenly distributed in the cell	Inside the cell
	Octamer bound to DNA	120	0	Inside the nucleoid space	Stationary on DNA
	Tetramer	0	10	n/a	Inside the cell
	Dimer	0	10	n/a	Inside the cell
ArcB^3^	Protein molecule	1000	5	Inner membrane	Inner membrane
Cyo	Oxidase molecule	Vary at different AU levels^4^	5	Inner membrane	Inner membrane
Cyd	Oxidase molecule	Vary at different AU levels^4^	5	Inner membrane	Inner membrane

The third column lists the number of molecules used to initiate the model.

1 See Table 4.

2 The ArcA numbers were reported by Rolfe et al. [3].

3 The ArcB numbers are assumed based on the ∼10∶1 ratio of response-regulator to sensor-kinase for another *E. coli* two-component system PhoB-PhoR [22].

4 Numbers are listed in Table 1.

**Table 3:** Agent interaction rules.

Molecules	Interaction rules^1^
O_2_	Interacting with FNR dimer, ArcB, Cyo and Cyd
FNR dimer	*FNR dimer+O_2_* 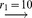 *FNR monomer+FNR monomer*
	*FNR dimer+binding site* 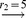 *FNR dimer bound to binding site*
FNR monomer	*FNR monomer+FNR monomer* 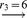 *FNR dimer*
FNR dimer bound to DNA	*FNR dimer bound to binding site+O_2_* 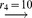 *FNR monomer+FNR monomer+unoccupied binding site*
ArcB	*ArcB∼P+O_2_* 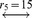 *ArcB+O_2_*
ArcA octamer	*ArcA octamer+ArcB* 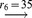 *ArcA tetramer+ArcA dimer∼P+ArcA dimer+ArcB∼P*
ArcA tetramer	*ArcA tetramer+ArcA tetramer * 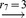 * ArcA octamer*
	*ArcA tetramer+ArcB * 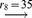 * ArcA dimer∼P+ArcA dimer+ArcB∼P*
ArcA dimer∼P	*ArcA dimer∼P+ArcA dimer∼P* 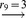 *ArcA tetramer*
	*ArcA dimer∼P+ArcB* 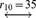 *ArcA dimer+ArcB∼P*
ArcA dimer	*ArcA dimer+ArcB∼P* 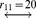 *ArcA dimer∼P+ArcB*
ArcA octamer bound to DNA	ArcA octamer bound to DNA is assigned a probability of 0.3% to leave the DNA in every iteration. This ‘off rate’ is required because ArcA∼P dephosphorylation occurs by the action of ArcB at the cell membrane (see text).
Cyo	*Cyo+O_2_* 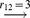 *Cyo+H_2_O*
Cyd	*Cyd+O_2_* 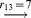 *Cyd+H_2_O*

The interaction radii (nm) were defined and refined as described in the *Methods*.

1 Additional descriptions of the ArcBA and FNR interaction rules are provided in the *Supporting Information*.

The algorithm of supplying O_2_ to the cell responds to given AU levels and automatically calculates the rate of O_2_ molecules supplied to the cell. Whilst the model runs, agent information is updated and stored as a series of XML files for further analysis and visualization.

### Modeling the regulatory response to O_2_ availability

**Figure 3:**
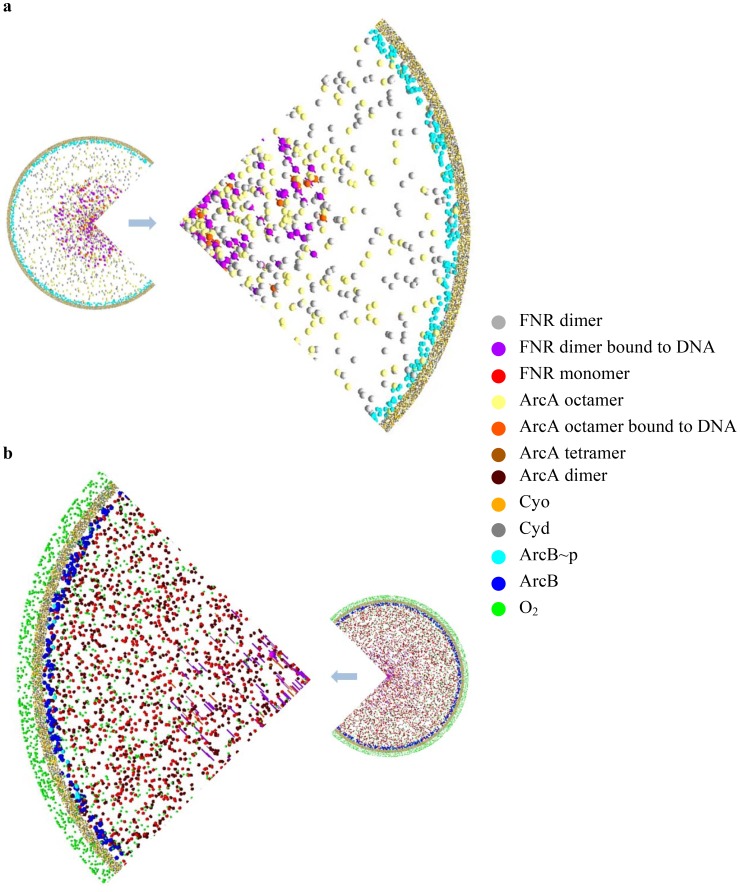
The initial and final states of model with no O_2_ and with excess O_2_ supplied. (a) The initial state of model with no O_2_ (0% AU) supplied to the cell. (b) The final state of model with excess O_2_ (217%) supplied to the cell. Each type of molecule is represented by a different color as shown in the key.

The dynamics of the system were investigated by running the simulation through two cycles of transitions from 0–217% AU. Figure 3a shows a top view of a 3-D *E. coli* cell at 0% AU (steady-state anaerobic conditions). Under these conditions, the FNR molecules are present as dimers, all ArcB molecules are phosphorylated and the ArcA is octameric. The DNA binding sites for ArcA (120 in the model) and FNR (350 in the model) in the nucleoid are fully occupied. The number of ArcA sites was chosen from the data reported by Liu and De Wulf [18]. The model must include a mechanism for ArcA∼P to leave regulated promoters. Upon introduction of O_2_ into anaerobic steady-state chemostat cultures ∼5 min was required to inactivate ArcA-mediated transcription [15]. In the agent-based model presented here, each iteration represents 0.2 sec. Therefore, assuming that ArcA∼P leaving the 120 DNA sites is a first order process, then t_½_ is ∼45 sec, which is equivalent to ∼0.3% ArcA∼P leaving the DNA per iteration (Table 3). The number of FNR binding sites was based on ChIP-seq and ChIP-Chip measurements, which detected ∼220 FNR sites and a genome sequence analysis that predicted ∼450 FNR sites; thus a mid-range value of 350 was chosen [23]–[25]. Interaction with O_2_ causes FNR to dissociate from the DNA (Table 3). Under fully aerobic conditions (217% AU) the FNR dimers are disassembled to monomers, and the different forms of ArcA coexist (Figure 3b). The ArcA- and FNR- DNA binding sites in the nucleoid are mostly unoccupied due to the lower concentrations of FNR dimers and ArcA octamers. Examination of the system as it transits from 0% to 217% AU showed that the DNA-bound, transcriptionally active FNR was initially protected from inactivation by consumption of O_2_ at the cell membrane by the terminal oxidases and by reaction of O_2_ with the iron-sulfur clusters of FNR dimers in the bacterial cytoplasm - the progress of this simulation is shown in Video S1. This new insight into the buffering of the FNR response could serve a useful biological purpose by preventing pre-mature switching off of anaerobic genes when the bacteria are exposed to low concentration O_2_ pulses in the environment.

**Figure 4:**
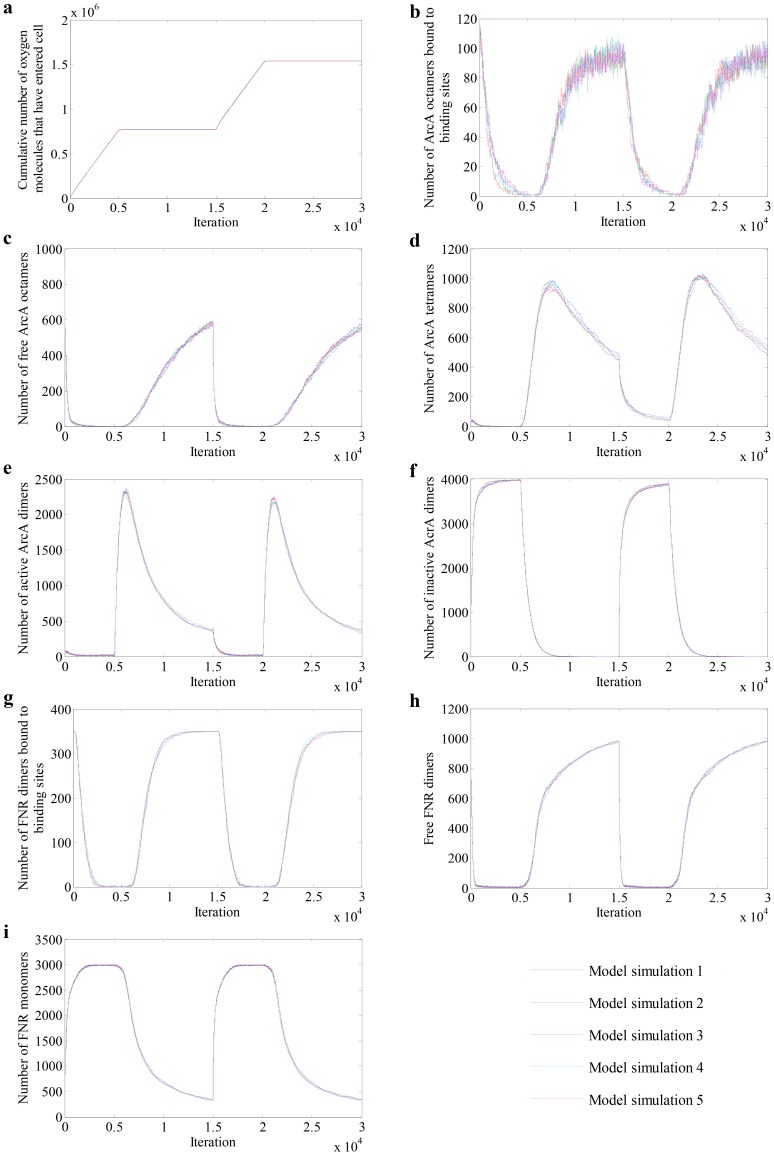
Model simulation output: quantitative variation of ArcA and FNR molecules in response to O_2_ availabilities. (a) Cumulative number of O_2_ molecules that have entered the modeled cell. (b–f) The changes in the numbers of ArcA octamers bound to DNA, free ArcA octamers, ArcA tetramers, active (phosphorylated) ArcA dimers, and inactive (de-phosphorylated) ArcA dimers. (g–i) The profiles of FNR dimers bound to DNA, free FNR dimers and FNR monomers. The data were taken from 5 repeats of model simulation with 30,000 iterations per run.

In the various niches occupied by *E. coli*, the bacterium can experience the full range of O_2_ concentrations from zero, in the anaerobic regions of a host alimentary tract, to full O_2_ saturation (∼200 µM, equivalent to ∼120,000 O_2_ molecules per cell), but fully aerobic metabolism is supported when the O_2_ supply exceeds 1,000 O_2_ molecules per cell. The profiles of five repetitive simulations for each agent in the model are presented in Figure 4. From iteration 1 to 5000 and iteration 15000 to 20000, O_2_ was supplied at a constant value of ∼6,500 molecules per cell such that the total number of O_2_ molecules entering the cell increased linearly; when the O_2_ supply was stopped (5000 to 15000 and 20000 to 30000 iterations) no more O_2_ entered the cell and thus the number of O_2_ molecules that had entered the cell remained unchanged during these periods (Figure 4a). When O_2_ became available to the cell (from iteration 1), the sensor ArcB was de-phosphorylated and started to de-phosphorylate ArcA. Consequently, the number of ArcA octamers bound at their cognate sites in the nucleoid decreased rapidly. The ArcA tetramers and dimers produced during de-phosphorylation of the ArcA octamer were transformed to inactive (de-phosphorylated) ArcA dimers, (Figure 4d–f). Under aerobic conditions (iteration 5000) all the ArcA was decomposed to inactive ArcA dimers. When the O_2_ supply was stopped (from iteration 5001), the number of inactive ArcA dimers decreased rapidly as shown in Figure 4f, being transformed into phosphorylated ArcA dimers, tetramers and octamers (Figure 4c–e). Due to the phosphorylated ArcA dimers and tetramers combining to form ArcA octamers, their numbers dropped after initially increasing. The rate at which the ArcA octomers accumulated (ArcA activation) after O_2_ withdrawal was slower than the rate of ArcA inactivation (Figures 4b and c). In this implementation of the modeled transition cycle, the numbers of ArcA octamers in the cytoplasm and bound to DNA did not reach that observed in the initial state before the second cycle of O_2_ supply began, indicating that a longer period is required to return to the fermentation state.

The numbers of FNR dimer bound to binding sites and free FNR dimer (cytoplasmic FNR dimer) decreased when O_2_ was supplied to the system (Figures 4g–h), but the rate was slower than that for ArcA inactivation, consistent with O_2_ consumption at the membrane, which can be sensed by ArcB to initiate inactivation of ArcA, but lowers the signal for inactivation of FNR. When O_2_ was removed from the system (from iteration 5001) FNR was activated over a similar timeframe to ArcA (Figures 4b and g), which was again consistent with previous observations [15]. As with ArcA, free FNR dimers and FNR monomers did not fully return to their initial states after O_2_ supply was withdrawn in the model, indicating that further iterations are required to reach steady-state (Figure 4h–i). These results clearly indicate that the model is self-adaptive to the changes in O_2_ availability, and the reproducible responses prove the reliability and robustness of the model. The ArcBA system simulated in this model is based on a preliminary biological assumption, and the agent-based model presented here should prove a reliable and flexible platform for exploring the key components of the system and testing new experimental findings.

### Model validation

In order to validate the model with biological measurements of FNR DNA-binding activity estimated using an FNR-dependent *lacZ* reporter, the ArcBA system agents were removed from the model by setting their agent numbers to zero. The ArcBA system is an indirect O_2_ sensor and does not consume O_2_, hence the FNR system was not affected by withdrawing ArcBA from the model, but this simplification increased simulation speed.

The O_2_ step length and other model parameters were estimated using the experimental data obtained at 31% AU. Using the estimated O_2_ step length at 31% AU and defining the step length of O_2_ molecule, 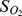, as 0 at 0% AU, a linear model, 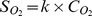, was constructed to predict the step lengths of O_2_ at other AU levels, where *k* = 2.1 and 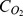 represents the O_2_ concentration at different AU levels (Table 4). The O_2_ step lengths predicted by this model were used to validate the model at 85%, 115% and 217% AU, and the accuracy of the linear model was shown by the good correlation between the model and experimental data.

**Table 4:** Relative parameters for O_2_ molecules.

AU (%)	O_2_ in input gas (%)	O_2_ molecules per cell per iteration	Step length of O_2_ molecule (nm)
0	0	0	0
31	2.8	13	6
85	8.1	37	14.5±2.5
115	11.1	51	25.5±0.5
217	21.1	97	41.5±1.5

The percentages of O_2_ in the input gas at different AU levels (first column) are listed in the second column. The third column presents the calculated numbers of O_2_ molecules supplied to a cell. The step length values are listed in the fourth column are obtained from a set of model tests at 31% AU only and were validated at 85%, 115% and 217% AU.

**Figure 5:**
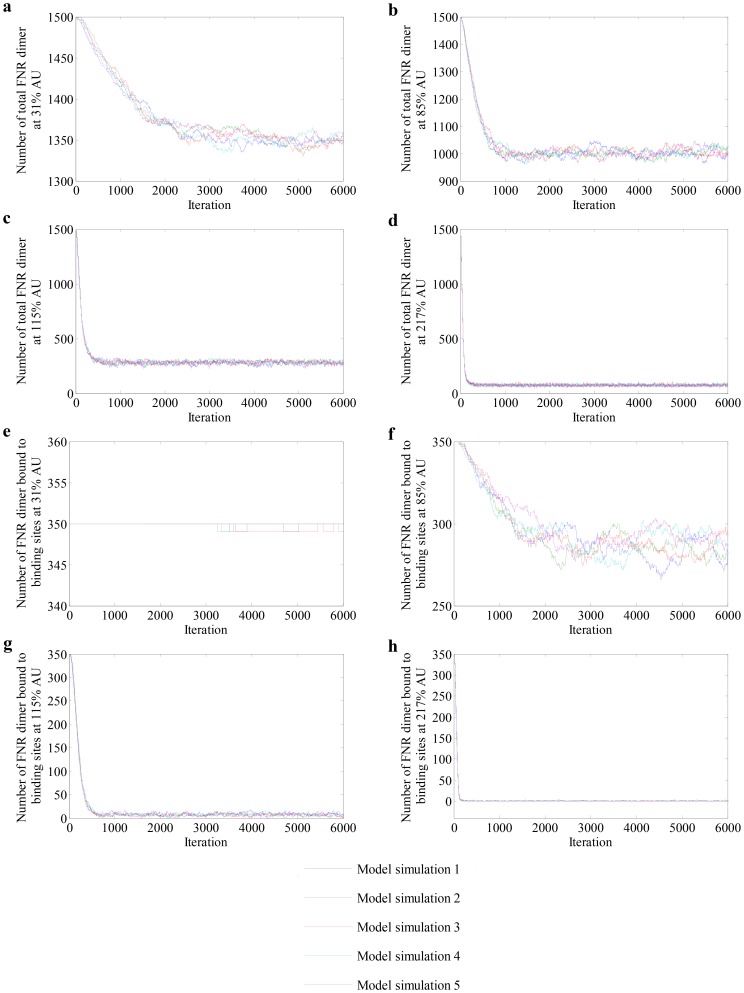
Profiles of FNR dimers at different AU levels. (a–d) Variation of the numbers of total FNR dimers and (e–h) numbers of FNR dimers bound to DNA at AU levels 31%, 85%, 115% and 217% respectively.

Profiles of five repetitive simulations in which the simplified model was used to predict the numbers of active FNR dimers in steady-state cultures of bacteria grown at different AU values are presented in Figure 5. At 31% AU, the model implied that FNR-mediated gene expression is unaffected compared to an anaerobic culture (0% AU), i.e. the number of FNR binding sites occupied in the nucleoid remained unchanged (Figures 5a and e). Even at 85% AU, ∼80% of the FNR-binding sites remained occupied (Figures 5b and f). It was only when the O_2_ supply was equivalent to >115% AU that occupation of the FNR-binding sites in the nucleoid decreased (Figures 5 c, d, g and h). These outputs matched the FNR activities calculated from the measurements of an FNR-dependent reporter (Table 5) and thus demonstrate the abilities of the model to simulate the general behavior of FNR dimers in steady-state cultures of *E. coli*.

**Table 5:** Comparison between experimental and simulation results for wild-type FNR.

	Simulation results			Experimental results	
Aerobiosis units (%)	Total FNR dimer (molecules per cell)	FNR dimer bound to binding sites (molecules per cell)	Predicted output from an FNR-dependent promoter (% of maximum)	Total FNR dimer (molecules per cell)^1^	Measured output from an FNR-dependent promoter (% of maximum)^2^
0	1500	350	100	1500	100±3
31	1350±6	350	100	1377	95±4
85	1033±16	287±16	82±4.6	1020	67±7
115	280±15	7±3	2±0.9	248	13±3
217	73±8	0	0	60	0

The model simulation data was taken by averaging the model outputs at steady-state. For these results, the values shown are the averages and standard deviations. The last column shows experimentally determined transcription from the FNR-dependent *FF-41.5-lacZ* promoter; the transcriptional output at 0% AU was set to 100.

1 The total numbers of FNR dimers were calculated from the Western blots and FNR-dependent promoter activities.

2 The measured output from an FNR-dependent promoter was reported by [15], [38].

A second validation approach using two FNR variants that are compromised in their ability to undergo monomer-dimer transitions was adopted. The FNR variant FNR I151A can acquire an iron-sufur cluster in the absence of O_2_, but subsequent dimerization is impaired [26]. The FNR D154A variant can also acquire an iron-sulfur cluster under anaerobic conditions, but does not form monomers in the presence of O_2_ [26]. To mimic the behavior of these two FNR variants the interaction radius for FNR dimer formation was changed in the model. Thus, the interaction distance for wild-type FNR monomers, which was initially set at 6 nm (r_3_, Table 3) was increased to 2000 nm for the FNR D154A variant, essentially fixing the protein as a dimer, or decreased to 2.5 nm for the FNR I151A variant, making this protein predominantly monomeric under anaerobic conditions. The results of simulations run under aerobic (217% aerobiosis) and anaerobic conditions (0% aerobiosis) suggested that under aerobic conditions wild-type FNR and FNR I151A should be unable to inhibit transcription from an FNR-repressed promoter (i.e. the output from the reporter system is 100%), whereas FNR D154A should retain ∼50% activity (Table 6). Under anaerobic conditions, wild-type FNR was predicted to exhibit maximum repressive activity (i.e. 0% reporter output), whereas FNR I151A and FNR D154A mediated slightly enhanced repression compared to the simulated aerobic conditions (Table 6). To test the accuracy of these predictions, the ability of wild-type FNR, FNR I151A and FNR D154A to repress transcription of a synthetic FNR-regulated promoter (*FFgalΔ4*) under aerobic and anaerobic conditions was tested [27]. The choice of a synthetic FNR-repressed promoter was made to remove complications that might arise due to iron-sulfur cluster incorporation influencing the protein-protein interactions between FNR and RNA polymerase; in the reporter system chosen FNR simply occludes the promoter of the reporter gene and as such DNA-binding by FNR controls promoter activity. The experimental data obtained matched the general response of the FNR variants in the simulation, but not very precisely for FNR D154A, with the experimental data indicating more severe repression by FNR D154A under both aerobic and anaerobic conditions than predicted (Table 6). This suggested that the interaction radius (r_2_ = 5 nm; Table 3), which controls the binding of FNR to its DNA target required adjustment to enhance DNA-binding of the FNR D154A variant. Therefore, the simulations were rerun after adjusting r_2_ to 7 nm for all the FNR proteins considered here. The results of the simulations for both FNR variants now matched the experimental data well (Table 6). However, it was essential to ensure that the adjustment to r_2_ did not significantly influence the model output for wild-type FNR. Therefore, simulations of the behaviour of wild-type FNR at 31, 85, 115 and 217% aerobiosis were repeated using the adjusted r_2_ value of 7 nm. The model output was very similar to those obtained when r_2_ was at the initial value of 5 nm (Table 7). These analyses imply that for FNR D154A, which is essentially fixed in a dimeric state, the rate of binding to the target DNA governs transcriptional repression, but for wild-type FNR the upstream monomer-dimer transition is the primary determinant controlling the output from the reporter.

**Table 6:** Comparison between experimental and simulation results for FNR variants.

Expression from an FNR-repressed promoter (%)						
Strain	Aerobic			Anaerobic		
	Simulation (r_3_ = 5 nm)^1^	Experiment^2^	Simulation (r_3_ = 7 nm)	Simulation (r_3_ = 5 nm)	Experiment	Simulation (r_3_ = 7 nm)
FNR	100	108.2±2.2	99.7	0	13.0±1.2	0
FNR I151A	100	126.7±2.8	100	87.5±1.4	83.3±4.4	79.6±0.7
FNR D154A	52.0±1.2	26.2±1.2	30.4±0.6	52.0±2.1	32.6±3	31.5±1.3

1 For the simulations, the standard deviations were calculated from 5 repeats for FNR and 13 repeats for FNR I151A and FNR D154A. The aerobic condition in simulation was modelled at AU level 217%, and the anaerobic condition was modelled at AU level 0%. r_3_ is the interaction radius between the FNR dimer and its cognate DNA-binding site.

2 For the experimental data 100% expression was set as the β-galactosidase activity obtained in the absence of FNR. Measurements were made from three independent cultures.

**Table 7:** The effect of interaction distance (r_3_) for binding of FNR dimers to target DNA on wild-type FNR activity.

	FNR dimer bound to binding sites (% of maximum)			
Interaction radius	31% AU	85% AU	115% AU	217% AU
r_3_ = 5 nm^1^	100	82±4.6	2±0.9	0
r_3_ = 7 nm	100	89.8±0.7	4.5±0.6	0.3

The standard deviations for r_3_ = 5 nm were obtained from 5 repeats and for r_3_ = 7 nm 10 repeats of the simulation.

1 r_3_ is the interaction radius between the FNR dimer and its cognate DNA-binding site.

### Concluding remarks

The FNR switch has been the subject of several attempts to integrate extensive experimental data into coherent models that account for changes in FNR activity and target gene regulation in response to O_2_ availability [15], [28]–[31]. These models have provided estimates of active and inactive FNR in *E. coli* cells exposed to different O_2_ concentrations and the dynamic behavior of the FNR switch. The ability of FNR to switch rapidly between active and inactive forms is essential for it to fulfill its physiological role as a global regulator and the models are able to capture this dynamic behavior. Thus, it is thought that the ‘futile’ cycling of FNR between inactive and active forms under aerobic conditions has evolved to facilitate rapid activation of FNR upon withdrawal of O_2_ and hence the physiological imperative for rapid activation has determined the structure of the FNR regulatory cycle [30], [31]. However, it is less clear from these approaches how the system avoids undesirable switching between active and inactive states at low O_2_ availabilities (micro-aerobic conditions, >0%–<100% AU). To achieve rapid FNR response times it has been suggested that minimizing the range of O_2_ concentrations that constitute a micro-aerobic environment, from the viewpoint of FNR, is advantageous [31]. Unlike previous models of the FNR switch, the agent-based model described here recognizes the importance of geometry and location in biology. This new approach reveals that spatial effects play a role in controlling the inactivation of FNR in low O_2_ environments. Consumption of O_2_ by terminal oxidases at the cytoplasmic membrane and reaction of O_2_ with the iron-sulfur clusters of FNR in the cytoplasm present two barriers to inactivation of FNR bound to DNA in the nucleoid, thereby minimizing exposure of FNR to micro-aerobic conditions by maintaining an essentially anaerobic cytoplasm for AU values up to ∼85%. It is suggested that this buffering of FNR response makes the regulatory system more robust by preventing large amplitude fluctuations in FNR activity when the bacteria are exposed to micro-aerobic conditions or experience environments in which they encounter short pulses of low O_2_ concentrations. Furthermore, investigation of FNR variants with altered oligomerization properties suggested that the monomer-dimer transition, mediated by iron-sulfur cluster acquisition, is the primary regulatory step in FNR-mediated repression of gene expression. It is expected that the current model will act as a foundation for future investigations, e.g. predicting the effects of adding or removing a class of agent to identify the significant regulatory components of the system.

## Methods

### Measurement of the rate of O_2_ supply

Knowledge of the rate of O_2_ supply, 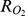, to the *E. coli* cells was required in order to simulate the response of the regulators of *cydAB* and *cyoABCDE* to different O_2_ availabilities. Therefore, un-inoculated chemostat vessels were used to measure dissolved O_2_ concentrations, 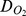, as a function of the percentage O_2_ in the input gas, *P_i_*, in the absence of bacteria. This allowed the rate at which O_2_ dissolves in the culture medium to be calculated from the equation: 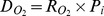, yielding 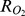 = 5.898 µmol/L/min. The number of O_2_ molecules distributed to a single bacterial cell was then calculated from the following equation: 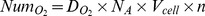 (where, *N_A_* is the Avogadro constant (6.022×10^23^), *V_cell_* is the volume of *E. coli* cell (0.3925 µm^3^) and as a constant for this equation, *n* (3.3×10^−9^) includes the unit transformations, min to sec (60^−1^) and µmol to mol (10^−6^), and the time unit represented by an iteration (0.2 sec).

### Control of agent mobility

**Figure 6:**
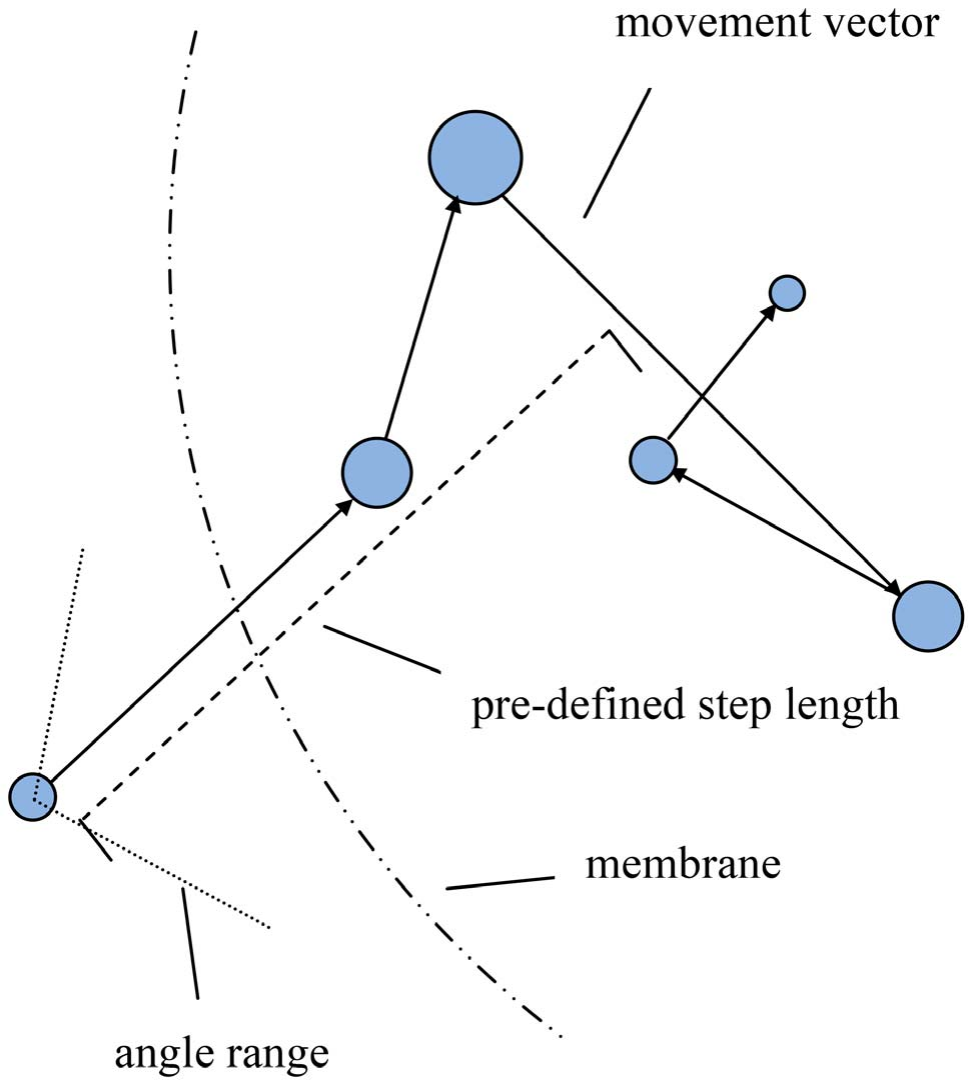
The 3-D movement of an O_2_ molecule during five successive iterations. Within a pre-defined limit (step length) the agent moves a random distance per iteration. For O_2_ this is also used to imitate the diffusion along the concentration gradient according to Fick's first law, in which the flux goes from regions of high concentration to regions of low concentration with a magnitude that is proportional to the concentration gradient (spatial derivative). The adjustment of step length of O_2_ (Table 4) affects the spatial moving speed, which is simply represented as the greater the step length, the faster the movement. The angle range was defined for O_2_ molecules that are outside the cell to enable them move towards cell. The O_2_ molecules inside the cell and all other molecules move in any direction within their defined spatial regions.

In the model the individual agents (Cyd, Cyo, ArcB, ArcA, FNR and O_2_) are able to move and interact within the confines of their respective locations in a 3-D-cylinder representing the *E. coli* cell. To control the velocity of agents, the maximal distances they can move in 3-D space during one iteration (step length) were pre-defined (Table 4). Thus, a step length is pre-defined in program header file (.h) and for each movement, this is multiplied by a randomly generated value within [0,1] to obtain a random moving distance, which in turn is directed towards a 3-D direction (movement vector) that was also randomly generated within defined spatial regions. An example is shown in Figure 6 to illustrate the movements of an O_2_ molecule when it enters the cell.

### Estimating interaction radii

Interactions between agents depend upon the biological rules governing their properties and being in close enough proximity to react. The interaction radius of an agent encapsulates the 3-D space within which reactions occur. As the interaction radii cannot be measured, they were first estimated on the basis of known biological properties. For the radii *r_1…4_*, *r_12_* and *r_13_* (Table 3), arbitrary values were initially set at 31% AU, and the model was then trained to match the experimental result for the number of FNR dimers at 31% AU (Table 5). The modeled output of FNR dimer number at steady-state was compared with the experimental data, and the difference suggested re-adjustment of interaction radii. The adjusted radii were then tested against the FNR dimer numbers at 85%, 115% and 217% AU (Table 5) during model validation, and the results indicate that the interaction radii values are capable of describing the behavior of the system. The interaction radii of Cyd and Cyo with O_2_ reflect their relative affinities for O_2_ (i.e. Cyd has a high O_2_ affinity and thus reacts more readily, 7 nm interaction radius, than Cyo, which has a lower affinity for O_2_, 3 nm interaction radius). As, thus far, no accurate biological data is available for ArcBA system, the radii *r_5…11_* were arbitrarily defined and were refined by training the model to match current biological expectations.

### Model description

The rod-shaped *E. coli* cell was modeled as a cylinder (500 nm×2000 nm) [32] with the nucleoid represented as a sphere with a diameter of 250 nm at the centre of the cell. The experimentally-based parameters and locations of the agents in their initial state are listed in Table 2. As the number of ArcB molecules has not been determined experimentally, this value was arbitrarily assigned (see above). The interaction rules for the agents are shown in Table 3 (additional descriptions of an exemplar agent (O_2_) and the rules for ArcBA and FNR are provided in , Table S1 and Text S1). These rules, combined with the interaction radii, determine the final status of the system. The scale of the model is such that high performance computers are required to implement it, and the flexible agent-based supercomputing framework, FLAME (http://www.flame.ac.uk) acted as the framework to enable the simulation [33], [34]. For more information on FLAME see Figure S2 and Text S2.

### Measurement of the activities of FNR and FNR variants *in vivo*

Plasmids encoding the FNR variants were constructed by site-directed mutagenesis (Quikchange, Agilent) of pGS196, which contains a 5.65 kb fragment of wild-type *fnr* ligated into pBR322 [35]. The three isogenic plasmids pGS196 (FNR), pGS2483 (FNR I151A) and pGS2405 (FNR D154A) were used to transform *E. coli* JRG4642 (an *fnr lac* mutant strain) containing a pRW50-based reporter plasmid carrying the *lac*-operon under the control of the *FFgal*Δ*4* promoter [27].

β-Galactosidase assays were carried out as described previously on strains grown in LBK medium at pH 7.2 containing 20 mM glucose [36], [37]. Cultures were grown either aerobically (25 ml culture in a 250 ml flask at 250 rpm agitation with 1∶100 inoculation) or anaerobically (statically in a fully sealed 17 ml tube with 1∶50 inoculation). Cultures (three biological replicates) were grown until mid-exponential phase (OD_600_ = 0.35) before assaying for β-galactosidase activity.

## Supporting Information

Figure S1 **Stategraph for O_2_ molecules.** In order to describe the model clearly, every agent is given a formal description to illustrate its states, memory, functions, and relevant messages that it sends out or receives from other agents (see Table S1). The stategraph for an oxygen agent is shown in the diagram.
(TIFF)

Figure S2 **Building a FLAME simulation file.** The agent definition (written in XMML) is parsed by a FLAME model parser, called *xparser*, which generates the simulation code. In the GCC environment, the code is compiled with the message board library, *libmboard*. The initial agent population settings are set in 0.xml file as the starting status of the model.
(TIFF)

Table S1 **Agent description for the O_2_ molecule.**
(DOCX)

Text S1 **Additional description of interaction rules for the regulatory systems, ArcBA and FNR.**
(DOCX)

Text S2 **The agent-based modeling framework: FLAME.**
(DOCX)

Video S1 **Simulation of ArcBA and FNR activities in response to O_2_ over two 0–217% AU cycles.**
(MP4)
