# Supplementary figures and images for: Agent-Based Modeling of Oxygen-Responsive Transcription Factors in Escherichia coli
Source: PLoS Comput Biol. 2014 Apr 24;10(4):e1003595. doi: 10.1371/journal.pcbi.1003595 (PMC3998891; doi:10.1371/journal.pcbi.1003595)

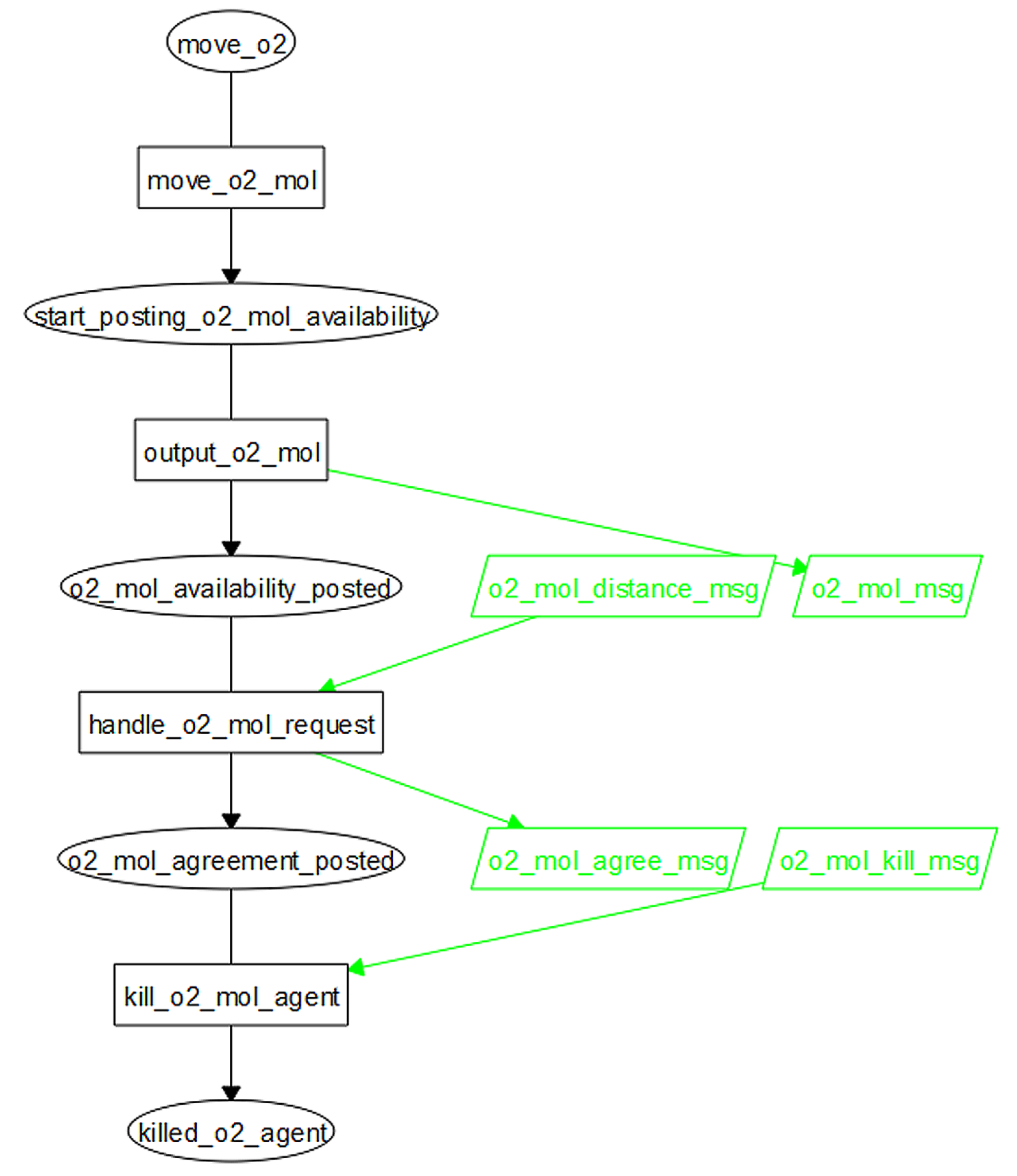

Supplement: Figure S1 — Stategraph for O2 molecules. In order to describe the model clearly, every agent is given a formal description to illustrate its states, memory, functions, and relevant messages that it sends out or receives from other agents (see Table S1). The stategraph for an oxygen agent is shown in the diagram. (TIFF) [file pcbi.1003595.s001.tiff]

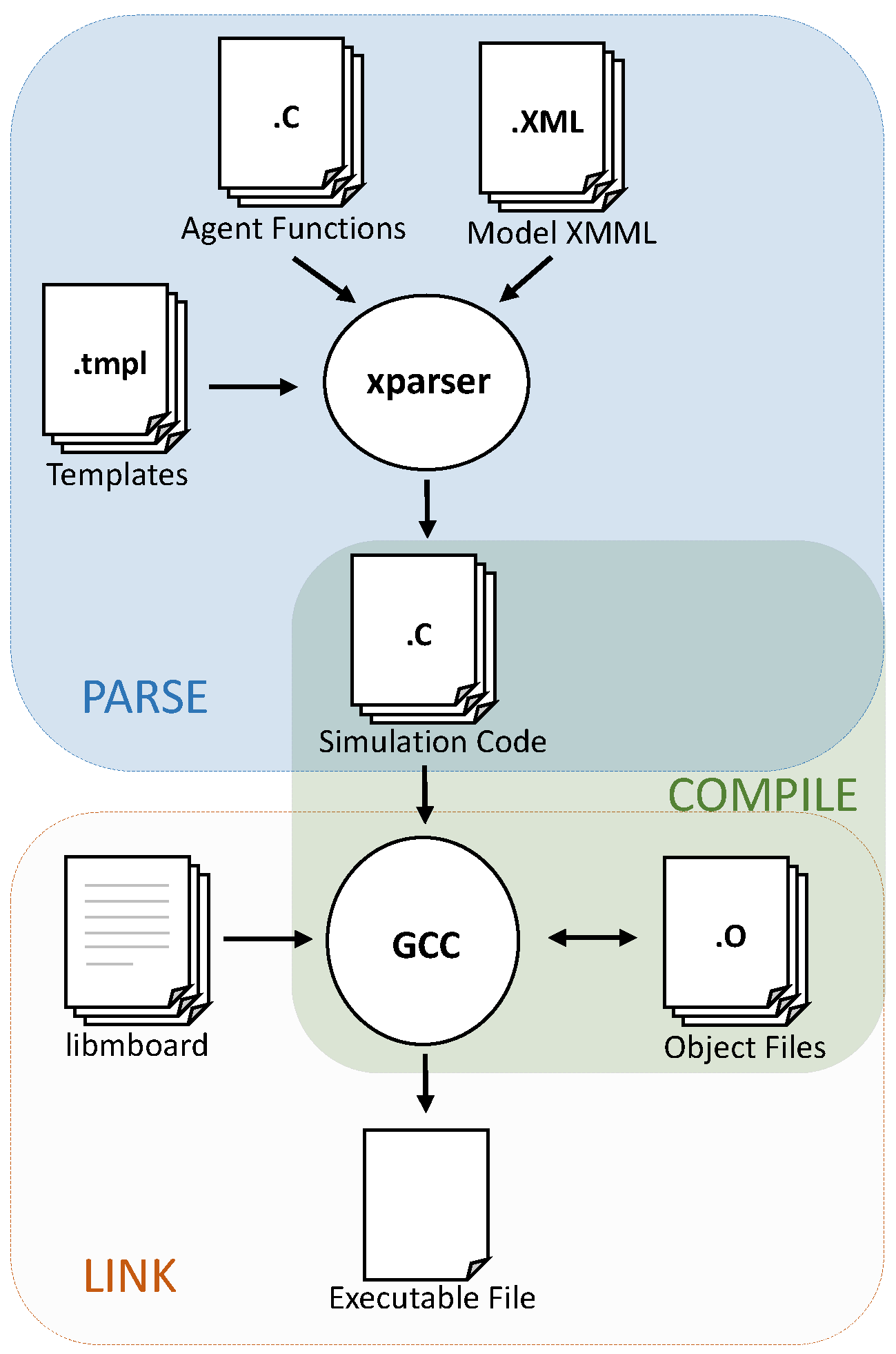

Supplement: Figure S2 — Building a FLAME simulation file. The agent definition (written in XMML) is parsed by a FLAME model parser, called xparser, which generates the simulation code. In the GCC environment, the code is compiled with the message board library, libmboard. The initial agent population settings are set in 0.xml file as the starting status of the model. (TIFF) [file pcbi.1003595.s002.tiff]
