# Supplementary material for: Agent-Based Modeling of Oxygen-Responsive Transcription Factors in Escherichia coli
Source: PLoS Comput Biol. 2014 Apr 24;10(4):e1003595. doi: 10.1371/journal.pcbi.1003595 (PMC3998891; doi:10.1371/journal.pcbi.1003595)
Supplement: Table S1 — Agent description for the O2 molecule. (DOCX) [file pcbi.1003595.s003.docx]

**Table S1. Agent description for the O_2_ molecule.** The O_2_ agent interacts with agents ‘FNR_mol’, ‘ArcA_dim_mol’, ‘ArcA_tet_mol’, ‘ArcA_oct_mol’, ‘ArcB_mol’, ‘Cyo_protein_mol’, and ‘Cyd_protein_mol’. By reading ‘o2_mol_distance_msg’ (see Figure S1) sent out by the above agents, the O_2_ molecule agent sends out ‘o2_mol_agree_msg’ to show its agreement of interaction. After a decision of interaction, the above agents (except ArcBA system agents) send out ‘o2_mol_kill_msg’. Upon receiving this message, the agent ‘o2_mol’ updates itself as killed, which means this agent will no longer exist.

| **Agent Memory** | | | | | | | | | | | | |
| --- | --- | --- | --- | --- | --- | --- | --- | --- | --- | --- | --- | --- |
| **Name** | | | | **Type** | | **Description** | | | | | | |
| o2_mol_id | | | | int | | ID for agent | | | | | | |
| o2_mol_agent_type | | | | int | | Defined as ‘6’ | | | | | | |
| o2_mol_x | | | | double | | Position x value | | | | | | |
| o2_mol_y | | | | double | | Position y value | | | | | | |
| o2_mol_z | | | | double | | Position z value | | | | | | |
| o2_mol_range | | | | double | | 1 | | | | | | |
| **Messages** | | | | | | | | | | | | |
| **Name :** | | | | **o2_mol_msg** | | | | | | | | |
| **Description:** | | | | Hold the information as defined for agent memory | | | | | | | | |
| **Elements** | | | | | | | | | | | | |
| **Name** | | | | **Type** | | **Description** | | | | | | |
| Id | | | | Int | | Information for current agent | | | | | | |
| agent_type | | | | Int | | Same as above | | | | | | |
| X | | | | double | | Same as above | | | | | | |
| Y | | | | double | | Same as above | | | | | | |
| Z | | | | double | | Same as above | | | | | | |
| Range | | | | double | | Same as above | | | | | | |
| **Name :** | | | | | | **o2_mol_agree_msg** | | | | | | |
| **Description:** | | | | | | Hold the information of the agent which satisfied with distance requirement for interaction | | | | | | |
| **Elements:** | | | | | | | | | | | | |
| **Name** | | | | **Type** | | **Description** | | | | | | |
| Id | | | | Int | | ID of agents interacting with o2 molecule | | | | | | |
| o2_mol_id | | | | Int | | ID of o2 molecule | | | | | | |
| **Functions:** | | | | | | | | | | | | |
| **Name :** | | | | **move_o2_mol** | | | | | | | | |
| **Description:** | | | | Move o2 molecule at start of iteration | | | | | | | | |
| **Current State:** | | | | **move_o2** | | | | | | | | |
| **Next State:** | | | | **start_posting_o2_mol_availability** | | | | | | | | |
| **Flow:** | | | | | | | | | | | | |
| Get current position of O_2_ molecule  ↓  Call ‘move_mol’ function to get new position data  ↓  Set new position values for current agent | | | | | | | | | | | | |
| **Inputs:** | | | | | | | | | | | | |
| **Message Name** | | | **Filter/operation** | | | | | | | | | **From Agent** |
| n/a | | | n/a | | | | | | | | | n/a |
| **Outputs:** | | | | | | | | | | | | |
| **Message Name** | | | | | | | **To Agent** | | | | | |
| n/a | | | | | | | n/a | | | | | |
| **Name :** | | | **output_o2_mol** | | | | | | | | | |
| **Description:** | | | Send availability and information of O_2_ molecule to message board | | | | | | | | | |
| **Current State:** | | | **start_posting_o2_mol_availability** | | | | | | | | | |
| **Next State:** | | | **o2_mol_availability_posted** | | | | | | | | | |
| **Flow** | | | | | | | | | | | | |
| Send ‘o2_mol_msg’ | | | | | | | | | | | | |
| **Inputs:** | | | | | | | | | | | | |
| **Message Name** | | | **Filter/operation** | | | | | | | | **From Agent** | |
| n/a | | | n/a | | | | | | | | n/a | |
| **Outputs:** | | | | | | | | | | | | |
| **Message Name** | | | | | | | **To Agent** | | | | | |
| o2_mol_msg | | | | | | | o2_comp; Cyd_protein_mol; Cyo_protein_mol; Fnr_mol; ArcB_mol | | | | | |
| **Name :** | | | **handle_o2_mol_request** | | | | | | | | | |
| **Description:** | | | Receive request for binding from other agents. | | | | | | | | | |
| **Current State:** | | | **o2_mol_availability_posted** | | | | | | | | | |
| **Next State:** | | | **o2_mol_agreement_posted** | | | | | | | | | |
| **Flow** | | | | | | | | | | | | |
| Read ‘o2_mol_distance_msg’  ↓  Sort distance table from closest to farthest  ↓  Send ‘o2_mol_agree_msg’ | | | | | | | | | | | | |
| **Inputs:** | | | | | | | | | | | | |
| **Message Name** | | **Filter/operation** | | | | | | | | | **From Agent** | |
| o2_mol_distance_msg | | a.o2_mol_id = m.o2_id  a message is accepted only if the o2_id of the message equals to current o2_mol agent id. | | | | | | | | | Fnr_Dimer_mol, Cyo_protein_mol, Cyd_protein_mol, ArcB_mol | |
| **Outputs:** | | | | | | | | | | | | |
| **Message Name** | | | | | | | | **To Agent** | | | | |
| o2_mol_agree_msg | | | | | | | | Fnr_Dimer_mol | | | | |
| **Name :** | | | | | **kill_o2_mol_agent** | | | | | | | |
| **Description:** | | | | | Kill the agent after interaction with other agents | | | | | | | |
| **Current State:** | | | | | **o2_mol_availability_posted** | | | | | | | |
| **Next State:** | | | | | **killed_o2_agent** | | | | | | | |
| **Flow:** | | | | | | | | | | | | |
| Read ‘o2_mol_kill_msg’  ↓  Kill the agent by returning ‘1’ | | | | | | | | | | | | |
| **Inputs:** | | | | | | | | | | | | |
| **Message Name** | **Filter/operation** | | | | | | | | | **From Agent** | | |
| o2_mol_kill_msg | a.o2_mol_id = m.o2_id  a message is accepted only if the o2_id of the message equals to current o2_mol agent id. | | | | | | | | | Fnr_Dimer_mol; Cyd_protein_mol; Cyo_protein_mol, ArcB_mol | | |
| **Outputs:** | | | | | | | | | | | | |
| **Message Name** | | | | | | | | | **To Agent** | | | |
| n/a | | | | | | | | | n/a | | | |
