# Supplementary material for: Agent-Based Modeling of Oxygen-Responsive Transcription Factors in Escherichia coli
Source: PLoS Comput Biol. 2014 Apr 24;10(4):e1003595. doi: 10.1371/journal.pcbi.1003595 (PMC3998891; doi:10.1371/journal.pcbi.1003595)
Supplement: Text S1 — Additional description of interaction rules for the regulatory systems, ArcBA and FNR. (DOCX) [file pcbi.1003595.s004.docx]

Text S1. Additional description of interaction rules for the regulatory systems ArcBA and FNR

The model described in the paper contains agents representing the: ArcA octamer, ArcA tetramer, ArcA dimer, ArcB and ArcA octamer bound to DNA. The ArcB molecules can be phosphorylated (ArcB~P; low O_2_) or dephosphorylated (ArcB; high O_2_) and consequently the ArcA dimers can also be phosphorylated or unphosphorylated. The phosphorylation state of the ArcBA system is governed by the following rules.

- When an O_2_ molecule is within pre-defined reaction distance to an ArcB~P, the ArcB~P will be de-phosphorylated and become able to capture phosphate from phosphorylated ArcA dimers, tetramers and octamers. When an ArcB has no O_2_ molecule to interact with, the dephosphorylating activity of ArcB is lowered proportionally by decreasing the reaction radius.
- When an ArcA octamer is close enough to react with ArcB, it will be de-phosphorylated and decompose to form one ArcA tetramer, one phosphorylated ArcA dimer and one dephosphorylated ArcA dimer.
- When an ArcA tetramer is close enough to react with ArcB, the ArcA tetramer will be de-phosphorylated and decompose to form one phosphorylated ArcA dimer and one de-phosphorylated ArcA dimer.
- When a phosphorylated ArcA dimer is close enough to react with ArcB, the ArcA dimer will be dephosphorylated.
- When a dephosphorylated ArcA dimer is close enough to react with ArcB(~P), the ArcA dimer will be phosphorylated.
- When two phosphorylated ArcA dimers are close enough to react they combine to form an ArcA tetramer;
- When two ArcA tetramers are close enough to react they combine to form an ArcA octamer.
- When the ArcA octamer is close enough to an available binding site, the ArcA octamer will bind to DNA.

There are three forms of FNR, the FNR monomer, FNR dimer and FNR dimer bound to DNA. The interactions between FNR and O_2_ are defined as:

- When an O_2_ molecule is within a pre-defined reaction distance to an FNR dimer, the FNR dimer is decomposed to two FNR monomers. If this dimer was bound to DNA, the binding site becomes unoccupied.
- When two FNR monomers are close enough to interact with, they combine to create an FNR dimer.
- When the distance between an FNR dimer and an unoccupied binding site is less than their interaction distance, the dimer binds to the DNA, and the status of this binding site switches to occupied.
