# Supplementary material for: Agent-Based Modeling of Oxygen-Responsive Transcription Factors in Escherichia coli
Source: PLoS Comput Biol. 2014 Apr 24;10(4):e1003595. doi: 10.1371/journal.pcbi.1003595 (PMC3998891; doi:10.1371/journal.pcbi.1003595)
Supplement: Text S2 — The agent-based modeling framework: FLAME. (DOCX) [file pcbi.1003595.s005.docx]

Text S2. The agent-based modeling framework: FLAME

The principle behind agent-based modelling is that each interacting component in a complex system is represented as an autonomous ‘agent’, a software artefact that is programmed to behave exactly as the component does in terms of its interactions with its environment and all the other components in the system and internally it behaves as the component does under all the feasible circumstances. A number of software environments exist for agent-based modeling, but the scale of many of these models, including the one described here, is such that high performance computers are required and there is only one robust option, flexible agent-based supercomputing framework (http://www.flame.ac.uk), that can be easily used. FLAME is an agent-based modeling framework which enables the simulation target systems. In FLAME each agent is described in terms of a set of internal states that it may go through, together with a description of its ‘internal’ memory containing important information about the individual agents including its precise location, state and direction of movement etc. As well as this, the model defines the sort of messages that agents can send which will convey information about their position, state etc. to other agents in the system. The agents will then behave according to a set of rules – functions – that determine what it will do under the current conditions – location, state, messages received etc. that the agents experiences at that time. The simulation then visits every agent in a random order and updates the complete system during one iteration. The total behavior of the system then emerges as a result of myriad interactions of the agents. A formal description of a representative agent is provided by Table S1. The process of building an executable FLAME simulation file is illustrated in Figure S2. The code generated can run on high performance computers (HPCs) and GPUs.

Due to the high demand of computation power, the FLAME model described here was run on a HPC, *Iceberg*. As a node of the White Rose Computing Grid, the *Iceberg* is the name of the Linux-based high performance computing cluster at Sheffield University, which provides ‘cloud computing’ with its INTEL-based cluster (912 INTEL cores, 8 Nvidia Tesla Fermi M2070 GPU units for GPU programming and 1920 GB memory) and AMD-based cluster (632 AMD cores and 2528 GB memory).
